# Acute Effects of the Psychedelic Phenethylamine 25I-NBOMe in C57BL/6J Male Mice

**DOI:** 10.3390/ijms26062815

**Published:** 2025-03-20

**Authors:** Sabrine Bilel, Cristina Miliano, Giorgia Corli, Marta Bassi, Massimo Trusel, Raffaella Tonini, Maria Antonietta De Luca, Matteo Marti

**Affiliations:** 1 Department of Translational Medicine, Section of Legal Medicine and LTTA Centre, University of Ferrara, 44121 Ferrara, Italy; sabrine.bilel@unife.it (S.B.); giorgia.corli@unife.it (G.C.); marta.bassi@unife.it (M.B.); 2 Department of Biomedical Sciences, University of Cagliari, 09042 Cagliari, Italy; cristinamiliano@hotmail.it; 3 School of Neuroscience, Virginia Polytechnic Institute and State University, Blacksburg, VA 24061, USA; 4 Neuromodulation of Cortical and Subcortical Circuits Laboratory, Neuroscience and Brain Technologies Department, Istituto Italiano di Tecnologia, 16163 Genova, Italyraffaella.tonini@iit.it (R.T.); 5 Collaborative Center for the Italian National Early Warning System, Department of Anti-Drug Policies, Presidency of the Council of Ministers, 00186 Roma, Italy

**Keywords:** 5HT_2A_ agonist, 25I-NBOMe, dopamine transmission, microdialysis, behavioral tests, prepulse inhibition, synaptic plasticity, LTP, LTD

## Abstract

25I-NBOMe (4-Iodo-2,5-dimethoxy-N-(2-methoxybenzyl) phenethylamine) is a synthetic psychedelic compound abused for its ambiguous legal state as a counterfeit lysergic acid diethylamide (LSD). 25I-NBOMe acts as a selective agonist of 5HT_2A_ receptors leading to hallucinations, intoxications, and fatalities. Here, we assessed the rewarding properties of 25I-NBOMe and its behavioral and neurotoxic acute effects on the central nervous system of C57BL/6J mice. We evaluated the dopamine (DA) levels using in vivo microdialysis in the nucleus accumbens (NAc) shell after 25I-NBOMe (0.1–1 mg/kg i.p.) injection. We also investigated the effects of 25I-NBOMe (0.1–1 mg/kg i.p.) on locomotor activity, reaction time, and prepulse inhibition. Moreover, we assessed the acute 25I-NBOMe (1 µM) effects on synaptic transmission and plasticity in the medial prefrontal cortex (mPFC) by using ex vivo electrophysiology. Our findings suggest that 25I-NBOMe affects the DA transmission in NAc shell at the highest dose tested, increases the reaction time within 30 min after the administration, and disrupts the PPI. In slices, it prevents long-term synaptic potentiation (LTP) in the mPFC, an effect that could not be reverted by the co-administration of the selective 5HT_2A_ antagonist (MDL100907). Overall, these findings provide valuable new insights into the effects of 25I-NBOMe and the associated risks of its use.

## 1. Introduction

In the last decades, New Psychoactive Substances (NPSs) entered the drug market, and completely transformed the trends and patterns of use worldwide, posing new threats to public health [1,2]. The key features making these compounds so appealing to users of all ages are their easy accessibility via internet and their ambiguous legal status [3]. Initially designed and studied in research labs [4,5,6], many of these compounds were later adapted by criminal organizations, with drug designers developing a range of new substances capable of mimicking the effects of known illegal drugs across various chemical classes (cannabinoids, psychostimulants, cathinones, hallucinogens, and opioids) [7]. To achieve this, they modified the chemical structure to increase receptor affinity [8,9,10,11], creating “undetectable super drugs” that have led to numerous intoxications and fatalities [2,12,13]. Indeed, drug control institutions are facing unprecedented challenges due to the problematic analytical identification, and the rapid replacement of drugs scheduled by the authorities with new legal compounds [14]. Among all classes of NPSs, phenethylamines are synthetic psychedelic compounds abused mainly for their “entactogenic effects” [15], meaning enhanced sociability and empathy. Enjoying the psychedelic effects of substances is not a new trend, but according to the European Drug Agency (EUDA), 163 new hallucinogens (including 106 phenethylamines) were monitored in 2022 [16], and the total number of controlled hallucinogens is constantly increasing [17]. N-benzylmethoxy-substituted 2C hallucinogens (NBOMes) have been abused and used as radiolabeled tracers to map the brain serotoninergic receptors’ distribution as well [18,19]. 25I-NBOMe (4-Iodo-2,5-dimethoxy-N-(2-methoxybenzyl) phenethylamine), called “N-Bomb” or “Smiles” in slang, belongs to this class of phenethylamines and was sold as counterfeit LSD or MDMA (3,4-methylenedioxymethamphetamine) until it was scheduled as an illicit drug [20]. A big number of intoxications and fatalities were caused by this compound, mostly because of its toxic effects combined with an unawareness of its chemical composition and the relative doses of the recreational drug ingested [21,22]. Indeed, during a two-years period (2012–2014), a total of 138 exposure cases were reported [23]. Moreover, only in the United States, between 2016 and 2018 more than 600 seizures of 25I-NBOMe have been reported [24]. Additionally, a recent study featuring a series of case reports on 25I-NBOMe, conducted between 2014 and 2020 [25], emphasized that adverse effects can continue for months after the last use.

This compound has subnanomolar affinity for 5HT_2A_ receptors [26,27,28], and therefore small doses (500–800 µg) are consumed sublingually, sometimes by insufflation or intravenously, to obtain effects lasting up to 10 h [29]. Additionally, 25I-NBOMe displays micromolar affinity for several other receptors such as adrenergic (α1 and α2) and dopaminergic receptors (D1, D2, and D3), as well as monoamine transporters (NET, DAT, and SERT) [28,30]. It is also noteworthy that in vitro studies demonstrated that 25I-NBOMe activates the µ opioid receptor [31,32]. The inhibition of the monoamine reuptake transporters might be responsible for the MDMA-like effects, while the high affinity for receptors involved in the sympathetic nervous system can explain side effects like agitation, tachycardia, hypertension, heart failure, seizures, rhabdomyolysis, and acute kidney injury [33,34,35,36,37,38]. Furthermore, an excessive activation of the serotoninergic system might lead to symptoms such as tremors, diarrhea, neuromuscular rigidity, and hyperthermia—the so-called serotoninergic syndrome [39,40]. Reassuringly, the analytical detection of this compound and its congeners has made much progress in the identification of multiple NBOMes in a single blotter paper [41,42] and in biological fluids as well [43,44,45]. Moreover, it has been demonstrated that all NBOMes have a very fast plasma clearance due to a large first-pass metabolism [46,47], suggesting that the metabolites might be toxic themselves and/or be responsible for drug−drug interactions with other drugs, both therapeutic and recreational [19].

In the past few years, several in vivo studies have been conducted to explore the pharmacology and toxicology of 25I-NBOMe in rodents, and all the results are summarized in Table 1.

**Figure 1:**
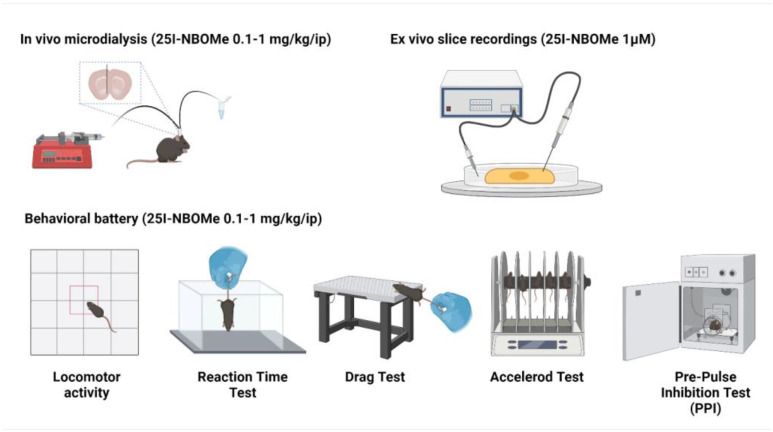
Schematic of the experiments performed. Created with BioRender.com (URL accessed on 22 October 2024).

The rewarding properties and related abuse liability of this compound have been demonstrated to some extent in rats, but not completely in mice. Hence, in this study we evaluated the effects of 25I-NBOMe on the dopaminergic transmissions in the ventral striatum, by using in vivo microdialysis. Moreover, as previously performed by our research team [56,57,58], we included in our preclinical study specific behavioral tests indicative of the neurotransmission alterations occurring after the treatment. These tests also provide translatable data from the animal model to humans, with a focus on the hallucinogenic and psychotic effects of 25I-NBOMe [50]. Indeed, in addition to reward-related functions, dopamine signals within the ventral and dorsal striatum are implicated in the modulation of movement and locomotion in mammals [59]. NBOMe compounds and 25I-NBOMe itself were found to induce an alteration in the spontaneous locomotion of rodents [48,55], and some authors suggested possible interactions between DA and other systems (such as the GABAergic and cholinergic) as responsible for the observed effect [52,60]. On this basis, we evaluated the effect of 25I-NBOMe on both the spontaneous (through the open field test) and stimulated (through the drag and the rotarod tests) locomotion of mice. Moreover, given the high affinity at the 5HT_2A_ receptor of 25I-NBOMe, we evaluated its ability to induce a dispersive and hallucinogenic state in mice and to alter the sensorimotor gating. Hence, we measured the reaction time, as it serves as an indicator of altered consciousness in humans, informing us on the potential risks associated with the consumption of this compound while operating a vehicle [50,55]. Moreover, we conducted the PPI test, which provides valuable insights on the susceptibility to psychotic episodes that may occur after consumption of the compound [50,61,62,63]. Previous studies have shown that repeated exposure to substances like cocaine, methamphetamine, or MDPV reduces the excitability of cortical cells [64]. Additionally, drugs such as heroin, amphetamine, and cocaine impair long-term depression (LTD) and long-term potentiation (LTP) in various brain regions [65,66,67]. However, the acute effects of 25I-NBOMe on synaptic activity in any brain region have not yet been investigated; thus, we used ex vivo electrophysiology to assess the effects of this compound in the medial prefrontal cortex (mPFC), a brain area highly involved in hallucinogens-induced altered states of consciousness. A schematic of the experiments conducted in this study is illustrated in Figure 1.

## 2. Results

### 2.1. DA Transmission

**Figure 2:**
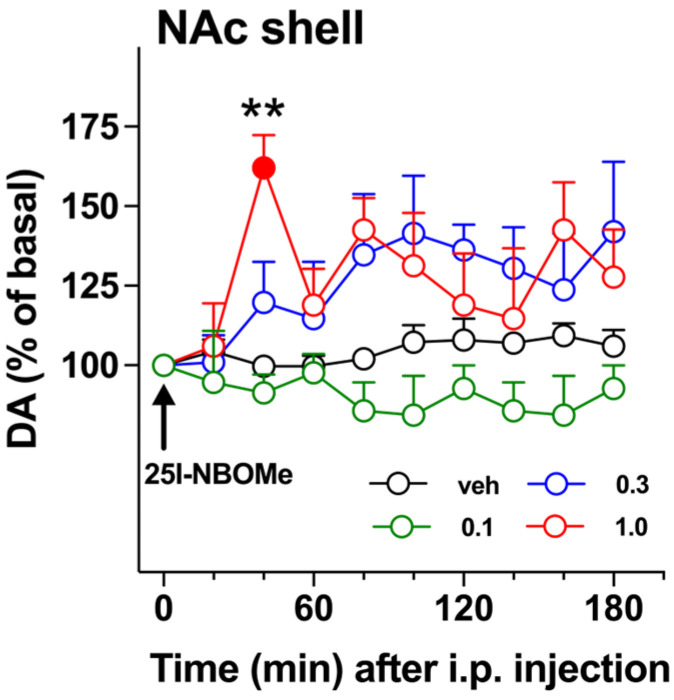
Effect of 25I-NBOMe administration (0.1–1.0 mg/kg i.p.) on DA transmission in male mice. Results are expressed as mean ± SEM of change in DA extracellular levels expressed as the percentage of basal values. The arrow indicates the start of i.p. injection of vehicle (black circles) or 25I-NBOMe 0.1 mg/kg (green circles), 0.3 mg/kg (blue circles), or 1.0 mg/kg (red circles) in the NAc shell. Statistical analysis was performed by two-way ANOVA followed by Tukey’s HSD post hoc test for multiple comparisons. Solid symbol: ** *p* < 0.01 with respect to basal values (vehicle N = 4; 0.1 mg/kg N = 4; 0.3 mg/kg N = 5; 1.0 mg/kg N = 4).

The effects of three doses of 25I-NBOMe (0.1, 0.3, and 1.0 mg/kg i.p.) on DA release was assessed in the NAc shell of freely-moving male C57BL/6J mice (Figure 2). The highest dose tested significantly affects DA transmission. Two-way ANOVA showed a main effect of treatment (F3,10 = 10.66; *p* < 0.01). Tukey’s post hoc tests showed differences at the 40 min sample from mice treated with 0.1 and 1.0 mg/kg i.p. One-way ANOVA of the latter group showed a main effect of treatment (F9,27 = 2.37; *p* < 0.05). Tukey’s post hoc tests showed a larger increase of dialysate DA at 40 min compared to the baseline.

### 2.2. Behavioral Tests

#### 2.2.1. Studies on Spontaneous Locomotor Activity in Mice

**Figure 3:**
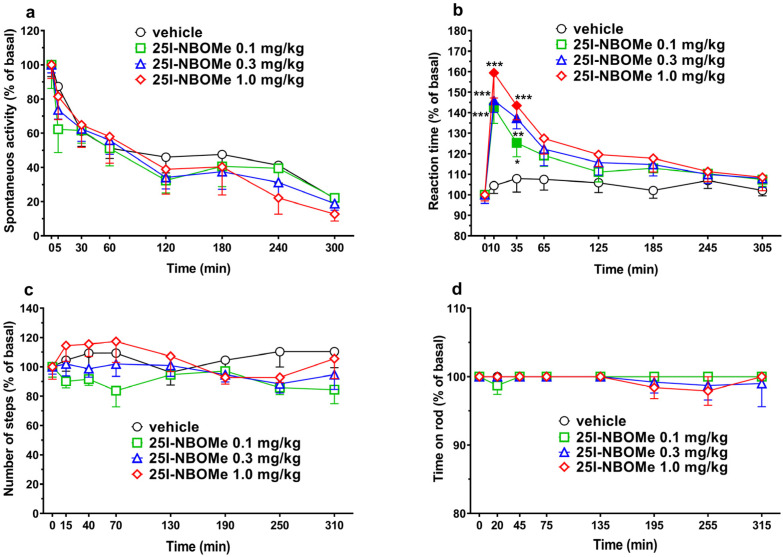
Effects of 25I-NBOMe (0.1–1 mg/kg i.p.) injections on the horizontal spontaneous motor activity (**a**), reaction time test (**b**), drag test (**c**), and accelerod test (**d**) in mice. Data are expressed as a percentage of the baseline (see Section 4) and represent the mean ± SEM of 8 determinations for each treatment. Statistical analysis was performed by two-way ANOVA followed by Bonferroni’s test for multiple comparisons. Solid symbol: * *p* < 0.05; ** *p* < 0.01; *** *p* < 0.001 versus vehicle.

Horizontal spontaneous locomotor activity tended to decrease in vehicle-treated mice over the 5 h observation (~60% of reduction at 300 min; Figure 3a).

The administration of 25I-NBOMe (0.1–1 mg/kg i.p.) did not affect the locomotor activity. Two-way ANOVA analysis showed an effect of time (F7,224 = 28.58, *p* < 0.0001).

#### 2.2.2. Reaction Time Test

The motor responsiveness of the animal in an open field after a fall remained unchanged in vehicle-treated mice over the 5 h observation. Systemic administration of 25I-NBOMe (0.1–1 mg/kg i.p.) dose-dependently increased the reaction time of the mice after falling but the transient effect persisted during the first hour after injection (Figure 3b). Two-way ANOVA analysis showed a significant effect of treatment (F3,224 = 16.40, *p* < 0.0001), time (F7,224 = 19.21, *p* < 0.0001), and time–treatment interaction (F21,224 = 2.003, *p* < 0.01).

#### 2.2.3. Drag Test and Accelerod Test

25I-NBOMe (0.1–1 mg/kg i.p.) affected neither the number of steps performed with the front legs of the mice (Figure 3c) nor the performance at the accelerod test (Figure 3d), during the 5 h of observation.

#### 2.2.4. Prepulse Inhibition Studies

**Figure 4:**
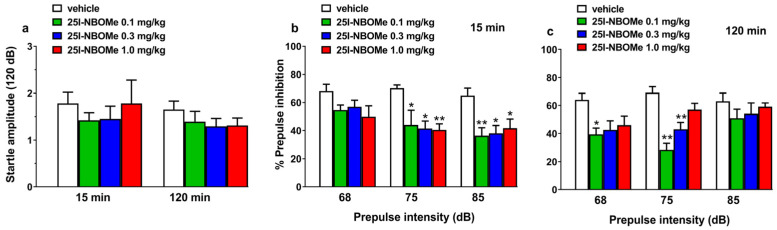
Effects of 25I-NBOMe (0.1–1 mg/kg, i.p.) on startle amplitude (**a**) and prepulse inhibition (PPI) in mice. Effects on PPI are shown for the three prepulse intensities (68, 75, and 85 dB), 15 min (**b**) and 120 min (**c**) after treatment. Prepulse inhibition (PPI) was expressed as the percentage decrease in the amplitude of the startle reactivity caused by presentation of the prepulse (%PPI) and values represent the mean ± SEM of 10 animals for each treatment. * *p* < 0.05; ** *p* < 0.01 versus vehicle.

Vehicle administration did not affect startle amplitude and PPI in mice. 25I-NBOMe injection (0.1, 0.3, and 1 mg/kg i.p.) did not affect startle amplitude in mice either at 15 or 120 min following the administration (Figure 4a).

However, 25I-NBOMe inhibited the PPI in mice at 15 min (Figure 4b), as shown by a significant effect of prepulse intensity at 75 dB (F3,39 = 4.921, *p* < 0.01) and 85 dB (F3,39 = 2.335, *p* < 0.005) detected by repeated measures ANOVA analysis; at 120 min (Figure 4c), ANOVA analysis detected a significant effect at 68 dB (F3,39 = 3.823, *p* < 0.05) and 75 dB (F3,39 = 14.46, *p* < 0.0001) of prepulse intensity but no effect with 85 dB.

### 2.3. Extracellular Field Recordings of Synaptic Activity in the Medial Prefrontal Cortex (mPFC)

**Figure 5:**
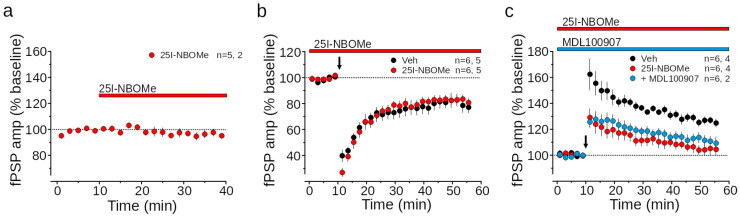
Effect of 25I-NBOMe on basal synaptic response and synaptic plasticity (**a**–**c**). The arrow indicates the application of 25I-NBOMe. Time courses of extracellular field post-synaptic potentials (fPSP) in the LV of mPFC (**a**) 30 min bath application of 25I-NBOMe (1 µM, red bar) did not affect fPSP amplitude (red circles, one-way ANOVA repeated measures F4,19 = 1.45, *p* > 0.05). (**b**) LTD was maintained in the presence of 25I-NBOMe (vehicle, black circles; 25I-NBOMe, red circles; vehicle versus 25I-NBOMe, Two-way ANOVA repeated measures, F1,10 = 0.14, *p* > 0.05). (**c**) Bath application of 25I-NBOMe impaired LTP induction. This effect was not prevented by 5HT_2A_ antagonism (vehicle, black circles; 25I-NBOMe, red circles; 25I-NBOMe + MDL100907, blue circles; Two-way ANOVA repeated measures, F2,14 = 15,11, *p* < 0.001, vehicle versus 25I-NBOMe, post-hoc Bonferroni, *p* < 0.001; vehicle versus 25I-NBOMe + MDL100907, post-hoc Bonferroni *p* < 0.01; 25I-NBOMe versus 25I-NBOMe + MDL100907, post-hoc Bonferroni *p* > 0.05). Conditioning protocols were delivered to the black arrow. Data are presented as time courses (mean ± s.e.m.) of normalized fPSP amplitudes.

Based on the reaction time and PPI results, the compound conceivably induces a dispersive and hallucinogen state, which is linked to alteration within the mPFC [68]. On this basis, we decided to investigate the effect of 25I-NBOMe synaptic plasticity in mPFC, rather than in the ventral striatum, on which future studies may be focused. To assess the functional effect of 25I-NBOMe on medial prefrontal cortex (mPFC) activity, we tested its impact on synaptic transmission and plasticity in acutely dissected brain slices. Extracellular field recordings of field post-synaptic potentials (fPSP) were conducted in cortical layer V upon the stimulation of axon fibers in layer II. We found that the bath application of 25I-NBOMe (1 µM) did not alter the fPSP amplitude (96.1 ± 2.0% of baseline, N = 5; *p* > 0.05; Figure 5a).

Next, we evaluated the compound’s effect on long-term depression (LTD) induced by low-frequency stimulation (LFS, 10 Hz, 10 min) on layer II [69]. This protocol resulted in a sustained (>30 min) depression of fPSP responses in vehicle-treated slices (DMSO 0.01%, 80.5 ± 4.7% of baseline, N = 6, *p* < 0.01; Figure 5b). Bath application of 25I-NBOMe did not affect LTD induction (82.5 ± 2.9% of baseline, N = 6, *p* < 0.05; Figure 5b).

We also assessed the effect of 25I-NBOMe application on long-term synaptic potentiation (LTP) induced by the high-frequency stimulation of LII projections to LV (HFS, 5 × 100 Hz, spaced by 10 s). This protocol reliably induced LTP of fPSP responses in slices superfused with vehicle (127.0 ± 2.7% of baseline, N = 6, *p* < 0.01; Figure 5c), but failed to induce LTP upon 25I-NBOMe application (105.1 ± 3.9% of baseline, N = 6, *p* > 0.05; Figure 5c). As 25I-NBOMe acts as a 5HT_2A_ receptor agonist [70], we tested whether 5HT_2A_ activation underlies the 25I-NBOMe-mediated impairment of LTP. Co-application of 25I-NBOMe with the 5HT_2A_ antagonist MDL100907 (300 nM) did not restore LTP (111.6 ± 4.1% of baseline, N = 6, *p* > 0.05; Figure 5c).

## 3. Discussion

The synthetic hallucinogen 25I-NBOMe has been used for its ambiguous legal status as a substitute LSD, and methamphetamine as well [71,72]. In this study, we evaluated the effects of 25I-NBOMe on the dopamine levels in the shell of NAc, and different behavioral tests such as locomotor activity, reaction time, and PPI. Furthermore, we investigated the acute neurotoxic effects of this compound by measuring its synaptic activity in the medial prefrontal cortex. Our results show that 25I-NBOMe increased the extracellular release of DA in the shell of the NAc. Behavioral data showed that this compound does not alter locomotor activity, while it impairs the reaction time at all doses tested and disrupts the prepulse inhibition at all doses at 15 and 120 min after the injection. In ex vivo slices, it irreversibly blocked the LTP in the mPFC.

### 3.1. 25I-NBOMe Influences the Dopaminergic Transmission in the NAc Shell

Our data showed that the highest dose (1 mg/kg i.p.) significantly increased the DA levels in the NAc shell, reaching 60% over the baseline at 40 min following the administration, and this effect lasted up to 80 min. The medium dose (0.3 mg/kg i.p.), however, did not show a high spike at 40 min; it showed a delayed effect by increasing the DA levels later and maintaining these levels up to 3 h after the administration. The lowest dose did not affect the DA transmission. Historically, an increase in the DA release in the ventral striatum has been associated with the rewarding properties of substances of abuse [72,73,74]; therefore, these data support the idea that this compound may display abuse liability. Moreover, the reinforcing properties of this compound have been recently demonstrated by using a conditioned placed preference paradigm in male mice at the dose of 0.3 mg/kg i.p. [54]. These findings agree with our previous study, where we showed that 25I-NBOMe (0.3 mg/kg i.p.) enhanced DA levels in the NAc shell in male rats 20 min after its administration [50]. However, the mice DA levels were not affected by this dose, while we observed a DA increase at the highest dose of 1 mg/kg i.p.; this discrepancy between species might be related to differences in the metabolism of this compound, and further investigations are required to explore this aspect. Our results also support the increase in DA striatal levels that Jeon and colleagues [49] observed ex vivo.

### 3.2. 25I-NBOMe Does Not Affect Locomotor Activity

Our data confirm previous studies showing that 25I-NBOMe up to 1 mg/kg i.p. does not affect spontaneous locomotion in mice [48]. Moreover, the administration of 25I-NBOMe at the tested doses does not impair either the stimulated motor activity or the motor coordination. These results agree with a previous study showing that 25I-NBOMe and its brominated analogue 25B-NBOMe cause motor inhibition at doses greater than 1 mg/kg [48]. This behavioral response is typically reported for other phenylalkylamine hallucinogens, such as 2C-C, 2C-D, 2C-E, 2C-I, 2C-T-2, DOC, and DOI [75,76,77], and it is mainly due to the stimulation of 5HT_2C_ receptors [46]. In our previous study with CD-1 mice, this compound (0.1 mg/kg i.p.) induced a transient facilitation of the spontaneous locomotion [55]; however, this discrepancy could be linked to the different strains employed. A similar effect was reported by Jo and colleagues with 25H-NBOMe [78]. Indeed, low doses of phenylalkylamine can increase locomotor activity by activating the 5HT_2A_ receptors [77]. In the present study, as reported in previous investigation [76], the 25I-NBOMe compound did not facilitated spontaneous locomotion at low doses.

### 3.3. 25I-NBOMe Impairs the Reaction Time Test and Disrupts the PPI

Similarly to what was previously observed [55], all tested doses of 25I-NBOMe induced in mice a significant increase in the reaction time (RT) test within 30 min after the administration. This effect could be related to the dispersive and hallucinatory effect of 25I-NBOMe [29], instead of a pure motor impairment as reported in DA-depleted mice [79]. In fact, drugs promoting serotonergic transmission, such as MDMA [63], or acting on the serotonergic receptor, such as 25I-NBOMe [50,55] and 2C-B [80], at low doses impair sensorimotor responses in rodents without affecting motor performance. In line with this, 25I-NBOMe up to 1 mg/kg induces a head twitch response (HTR) [29], and impairs sensorimotor functions [80] without reducing motor activity in mice (present data; [48,80,81]).

This study demonstrates that 25I-NBOMe reduces the prepulse inhibition (PPI) in male subjects, as previously reported in male mice and both male and female rats [50,55]. Several studies showed that phenylalkylamine and classical hallucinogens inhibit the PPI by stimulating the 5HT_2A_ receptors especially in the rat model, while in the mouse the involvement of this serotonergic receptor subtype seems marginal [77]. Therefore, further studies are needed to understand the involvement of specific serotoninergic receptor subtypes in the inhibition of sensorimotor gating induced by 25I-NBOMe in mice.

Additionally, low doses of 25I-NBOMe can alter the sensorimotor responses and sensory gating and induce HTS in mice without causing a direct reduction in motor activity. This profound sensory impairment could be the basis for the increase in reaction time in the RT test. Of note, more than 10 million cases of driving under the influence of drugs (DUID) were reported in 2016 alone [82], with the number of reported toxicological cases classified as DUID involving NPSs recently increasing from 28 (between 2019 and 2020; [83]) to 191 (between 2023 and 2024; [84]). Moreover, NBOMes drug-impaired driving has been reported as well [85]. Therefore, from a translational point of view in humans, low doses of 25I-NBOMe could cause sensory alterations capable of reducing response times in motor reactions and predisposing the individual to a greater risk in the case of driving vehicles or when performing dangerous activities in the workplace.

### 3.4. 25I-NBOMe Impairs LTP in the mPFC

Currently, limited data exist on the effects of psychedelics on PFC synaptic plasticity, a topic of interest given that drugs of abuse can disrupt mPFC functionality, thus impairing higher-order cognitive function [86,87,88]. Hence, we examined the effect of acute 25I-NBOMe treatment on LTD and LTP in the mPFC. Our findings indicate that while the compound did not affect fPSP or LTD, it significantly impaired LTP.

Previous research has shown that other psychedelics, like psilocybin, can enhance excitatory neurotransmission in the medial frontal cortex [89]. A recent study by de la Fuente Revenga et al. [90] reported that post-acute exposure to DOI, a phenethylamine sharing some structural similarity with 25I-NBOMe, enhanced cortical LTP. Another study reported that DOI induced LTD of evoked AMPA EPSCs in layer V pyramidal neurons and impaired electrically induced LTD in wild-type mice, but not in 5HT_2A_^−/−^ mice [91]. By contrast, amphetamine exhibits a biphasic effect, with low doses enhancing LTP in the PFC and higher doses impairing it [92].

Our results show that MDL100907, a selective 5HT_2A_ antagonist, did not reverse the LTD impairment, suggesting that 5HT_2A_ receptor activation is not implicated in this effect. Further studies are needed to elucidate the underlying mechanisms. Additionally, future research should explore whether higher doses of 25I-NBOMe may differently impact LTD and LTP.

## 4. Materials and Methods

### 4.1. Animals

Male C57BL/6J mice, 25–30 gr (ENVIGO, Italy; S. Pietro al Natisone, Italy), were group-housed on a reverse 12:12 h light-dark cycle, a temperature of 20–22 °C, and humidity of 45–55% and were provided ad libitum access to food (Diet 4RF25 GLP; Mucedola, Settimo Milanese, Milan, Italy) and water. All animal experiments were carried out in accordance with the Guidelines for Care and Use of Mammals in Neuroscience and Behavioral Research according to the Italian (D.Lgs 26/2014) and European Council Directive (2010/63/UE) and to the guidelines issued by the Committee for Animal Wellbeing (OPBA) at the University of Cagliari, the Animal Welfare Committee (OPBA) of the University of Ferrara, and the IIT of Genova, and were approved by the Italian Ministry of Health (license n. 223/2021-PR and extension CBCC2.46.EXT.21). Adequate measures were taken to minimize the number of animals used and animal pain and discomfort.

### 4.2. Drugs and Chemicals

25I-NBOMe was obtained from LGC Standards S.r.l. (Milan, Italy), and dissolved as previously described [50]. All doses (0.1, 0.3, 1.0 mg/kg) were injected through the intraperitoneal (i.p.) route of administration. For the electrophysiological recordings, NaCl, KCl, NaH_2_PO_4_, MgCl_2_, CaCl_2_, NaHCO_3_, ascorbic acid, and D-glucose were purchased from Sigma Aldrich; 25I-NBOMe was dissolved in DMSO and used at 1 µM concentration; the 5HT_2A_ antagonist MDL100907 was purchased from Tocris Biosciences (Bristol, UK), dissolved in DMSO and used at 300 nM concentration.

### 4.3. In Vivo Microdialysis

Male C57BL/6J mice, 25–30 g (ENVIGO, Italy; S. Pietro al Natisone, Italy) were anaesthetized with isoflurane (3% induction, 1–2% maintenance) and placed in a stereotaxic frame. During surgical procedures, the body temperature was checked and maintained at 36–38 °C with a controlled electric heating pad. The mice were implanted with a vertical dialysis probe (1 mm dialyzing portion) prepared as previously reported [50] with AN69 fibers (Hospal Dasco, Bologna, Italy) in the Nucleus Accumbens shell (NAc shell; A + 1.4, L 0.4 from bregma, V-4.8 from dura) according to the mouse brain atlas by Paxinos and Franklin (Second Edition, 2001). On the day following surgery, the probes were perfused with Ringer’s solution (147 mM NaCl, 4 mM KCl, 2.2 mM CaCl_2_) at a 1 μL/min flow rate. Dialysates were collected every 20 min (20 μL) and sampled into an HPLC (column -C18 3.5 um, Waters, Milford, MA, USA) coupled with a coulometric detector (ESA, Coulochem II, Chelmsford, MA, USA) for DA quantification as previously described [50]. The limit of detection for DA was 5 fmol/sample. Following the experiment, the mice were euthanized to collect brain tissue to verify the correct placement of the microdialysis probe.

### 4.4. Behavioral Studies

25I-NBOMe was studied using four behavioral tests widely employed in studies of “safety-pharmacology” for the preclinical characterization of new psychoactive substances [50,56,63,93]. To reduce the number of animals used, mice were evaluated in functional observational and behavioral tests carried out in a consecutive manner according to the following time scheme: spontaneous locomotion, evaluation of responsiveness of the animal in a reaction time test, and stimulated motor activity (drag and accelerod tests). The effect of 25I-NBOMe on startle/PPI response was investigated on a different group of mice. The experiments were performed between 9:00 a.m. and 1:00 p.m. For the overall behavioral study, 72 mice were used. Eight mice were used in the battery of behavioral tests of safety-pharmacology studies (spontaneous locomotion, reaction time, drag and accelerod tests) for each treatment (vehicle or 3 different 25I-NBOMe doses, 0.1, 0.3, and 1 mg/kg) (total mice used: 32); 10 mice were used in the startle/PPI analysis for each treatment (vehicle or 3 different 25I-NBOMe doses, 0.1, 0.3, and 1 mg/kg) (total mice used: 40).

#### 4.4.1. Spontaneous Locomotor Activity

Spontaneous locomotor activity was studied by using a camera (B/W USB Camera day&night with varifocal lens; Ugo Basile, Italy) and movies were analyzed off-line by a trained operator who did not know the drug treatments performed [58,63]. The mouse was placed in a square plastic cage (60 × 60 cm) located in a sound- and light-attenuated room and horizontal spontaneous motor activity was monitored and measured in seconds for 5 min at each time point (0, 5, 30, 60, 120, 180, 240, and 300 min). To avoid mice olfactory cues, cages were carefully cleaned with a dilute (5%) ethanol solution and washed with water between animal trials.

#### 4.4.2. Reaction Time Test Assessment

This test defines the motor responsiveness of the animal in an open field after a fall [55]. The mice were placed in the middle of an open square arena (15 × 15 cm) for 5 min to get used to the experimental context. Subsequently, they were gently lifted by the tail to a height of 3 cm from the surface of the arena and finally dropped, recording the time between the moment when the mouse touches the surface and the first movement of the limbs. The test was videotaped using a camera (B/W USB Camera day&night with varifocal lens; Ugo Basile, Italy) and movies were analyzed off-line by a trained operator who did not know the drug treatments performed. The frame-by-frame analysis allowed to evaluate the beginning of the reaction of the mouse while it moved its forelimbs to the floor for the first time. The reaction time test was performed at 0, 10, 35, 65, 125, 185, 245, and 305 min post-injection.

#### 4.4.3. Drag Test

The test measures the ability of the animal to balance its body posture with its front legs in response to an externally dynamic stimulus [81,94,95]. It provides information about the time that the mouse takes to start and run a movement (bradykinesia). The mouse was lifted by the tail, leaving the front paws on the table, and dragged backward at a constant speed of about 20 cm/s for a fixed distance (100 cm). The number of steps performed by each paw was recorded by two different observers. For each animal, we collected from five to seven measurements. The drag test was performed at 0, 15, 40, 70, 130, 190, 250, and 310 min post-injection.

#### 4.4.4. Accelerod Test

The test measures different motor parameters such as the motor coordination, the locomotive ability (akinesia/bradykinesia), the balance ability, the muscular tone, and the motivation to run. The animals were placed on a rotating cylinder that increased its velocity automatically in a constant manner (0–60 rotations/min over 5 min). The time spent on the cylinder was measured. The accelerod test was performed at 0, 20, 45, 75, 135, 195, 255, and 315 min post-injection [81,94,95].

#### 4.4.5. Prepulse Inhibition Analysis

The mice were tested for acoustic startle reactivity in startle chambers (Ugo Basile apparatus, Milan, Italy) consisting of a sound-attenuated, lighted, and ventilated enclosure holding a transparent non-restrictive Perspex^®^ cage (90 × 45 × 50 mm). A loudspeaker mounted laterally the holder produced all acoustic stimuli. The peaks and amplitudes of the startle response were detected by a loadcell. At the onset of the startling stimulus, 300 ms readings were recorded and the wave amplitude evoked by the mice startle response movement was measured [48,55,61].

Acoustic startle test sessions consisted of startle trials (pulse alone) and prepulse trials (prepulse + pulse). The pulse-alone trial consisted of a 40 ms 120 dB pulse. The prepulse + pulse trial sequence consisted of a 20 ms acoustic prepulse, an 80 ms delay, and then a 40 ms 120 dB startle pulse (100 ms onset–onset). There was an average of 15 s (range = from 9 to 21 s) between the trials. Each startle session began with a 10 min acclimation period with 65 dB broadband white noise that was present continuously throughout the session. The test session contained 40 trials composed of pulse-alone and prepulse + pulse trials (with three different prepulses of 68 dB, 75 dB, and 85 dB) presented in a pseudorandomized order. The animals were placed in the startle chambers 5 min after treatment with 25I-NBOMe. The entire PPI test lasted 20 min. The amount of prepulse inhibition (PPI) was expressed as the percentage decrease in the amplitude of the startle reactivity caused by the presentation of the prepulse (% PPI).

Startle/PPI responses were assessed at 15 and 120 min (including the 10 min acclimation period) after 25I-NBOMe i.p. injection.

### 4.5. Electrophysiological Study

The mice were anesthetized with isoflurane and decapitated, and their brains were transferred to ice-cold dissecting artificial cerebrospinal fluid (aCSF) containing 87 mM NaCl, 75 mM sucrose, 2.5 mM KCl, 1 mM NaH_2_PO_4_, 7 mM MgCl_2_, 25 mM NaHCO_3_, 10 mM D-glucose, and 0.5 mM ascorbic acid, saturated with 95% O_2_ and 5% CO_2_. Coronal sections (350 μm thick) were cut using a Vibratome 1000S slicer (Leica Biosystem, Deer Park, IL, USA), then transferred to aCSF containing 115 mM NaCl, 3.5 mM KCl, 1.2 mM NaH_2_PO_4_, 1.3 mM MgCl_2_, 2 mM CaCl_2_, 25 mM NaHCO_3_, and 25 mM D-glucose, and aerated with 95% O_2_ and 5% CO_2_. Following 20 min of incubation at 32 °C, slices were kept at 22–24 °C. During experiments, the slices were continuously superfused with aCSF at a rate of 2 mL min^−1^ at 28 °C, or aCSF containing either 25I-NBOMe (1 µM), or the 5HT2A antagonist MDL100907 (300 nM). Extracellular field recordings of field post-synaptic potentials (fPSPs) were obtained in layer V (L-V) of the limbic medial PFC, using glass micropipettes filled with aCSF. Stimuli were delivered via a constant-voltage isolated stimulator (Digitimer) through a bipolar twisted tungsten electrode placed in layer II (L-II) of the medial PFC. LTD was induced using the following low-frequency stimulation protocol (LFS): 10 Hz train for 10 min length [69]. LTP was induced by applying a high-frequency stimulation protocol (HFS) (5 × 100 Hz trains at 0.1 Hz). The stimulus intensity was set to evoke the half-maximal fPSP amplitude. Data were amplified and filtered (10 Hz to 3 kHz) by a differential amplifier (DAM 80 AC, World Precision Instruments, Sarasota, FL, USA), and digitized at 10 kHz (Digidata 1322, Molecular Devices, San Jose, CA, USA). fPSP amplitudes were measured using Axograph analysis software (https://axograph.com/, Axon Instruments, Foster City, CA, USA). The occurrence and magnitude of synaptic plasticity were evaluated by comparing the fPSP-normalized amplitudes from the last 5 min of baseline recordings with the values between 35–45 min after either LFS or HFS stimulation. Recordings in which the amplitude of the presynaptic fiber volley changed by more than 20% were discarded.

### 4.6. Data and Statistical Analysis

For the microdialysis experiments (Figure 2), data are expressed in percentages of basal DA values as mean ± SEM. One-way or repeated-measures ANOVA (two-way and three-way) were performed followed by Tukey’s tests for post hoc comparisons.

In the behavioral experiments (Figure 3; spontaneous locomotion, reaction time, drag and accelerod tests), data are expressed in percentages of basal values and are given as the mean ± SEM of 6–8 determinations for each treatment. Statistical analysis was performed using two-way ANOVA followed by Bonferroni’s test for multiple comparisons for the dose response curve of 25I-NBOMe at different times. In the startle/PPI experiments (Figure 4), the amount of PPI was calculated as a percentage score for each prepulse + pulse trial type: % PPI = 100 − {[(startle response for prepulse + pulse trial)/(startle response for pulse-alone trial)] × 100}. The startle magnitude was calculated as the average response to all the pulse-alone trials. For the ex vivo recordings (Figure 5), data were analyzed by utilizing one- or two-way ANOVA and Bonferroni post hoc comparison. All statistical analyses were performed by means of the Prism software 8.0 (GraphPad, San Diego, CA, USA).

## 5. Conclusions

In conclusion, our in vivo studies have shown that the acute administration of the psychedelic agent 25I-NBOMe: (1) dose-dependently increases DA release in the NAc shell, (2) dose-dependently increases the reaction time, (3) inhibits the PPI in adult C57BL/6J male mice, and (4) impairs the LTP in the mPFC. Therefore, the results of the present study improve our earlier characterization of the central effects of 25I-NBOMe [50], but also clarify the roles of the cortical and subcortical areas in the neurochemical and behavioral effects triggered by psychedelics, highlighting the need for great caution in their use. Further pharmacological and toxicological studies in female and adolescent mice can improve risk assessment and awareness to care for public health and safety.

## Figures and Tables

**Table 1:** Pharmacological studies about 25I-NBOMe.

Animal Model	Methods	Findings	Reference
C57BL/6J mice	25I-NBOMe (0.1–1 mg/kg; s.c.)	25I-NBOMe induced the head twitch response with 14-fold higher potency than 2C-I, and the effect was completely blocked by a selective 5HT_2A_ antagonist	[29]
Swiss-Webster mice	25I-NBOMe (1, 2.5, 5, 10, 25 mg/kg; i.p.)	25I-NBOMe decreased locomotor activity time-dependently and dose-dependently	[48]
Sprague-Dawley rats	25I-NBOMe (1, 2.5, 5, 10, 25 mg/kg; i.p.)	25I-NBOMe failed to produce 80% drug-appropriate responding in DOM-trained rats; in MDMA-trained rats 25I-NBOMe produced considerable fluctuations in drug-appropriate responding	
Sprague-Dawley rats	25I-NBOMe (0.01, 0.03, 0.1, 0.3 mg/kg; s.c.)	25I-NBOMe induced wet dog shakes and back muscle contraction, and the effect was blocked by a selective 5HT_2A_ antagonist	[27]
C57BL/6J mice	25I-NBOMe (0.3, 1, 2 mg/kg; i.p.)	25I-NBOMe increased conditioned place preference at the lowest dose tested	[49]
Sprague-Dawley rats	25I-NBOMe (0.03 mg/kg; inf)	25I-NBOMe did not induce intravenous self-administration	
Sprague-Dawley rats (male and female)	25I-NBOMe (0.3, 1 mg/kg; i.p.): microdyalisis 25I-NBOMe (0.1, 0.3, 0.5, 1.0 mg/kg; i.p.): behavior	Both sexes: 25I-NBOMe evoked increases in DA levels in the NAc shell, it caused impaired visual responses, and it disrupted the PPI. Females: core temperature was heavily affected. Males: the highest dose exerted an analgesic effect	[50]
Wistar-Han rats	25I-NBOMe (3 mg/kg; s.c.)	25I-NBOMe increased DA and 5HT in the frontal cortex	[51]
Wistar-Han rats	25I-NBOMe (1, 3 mg/kg; s.c.) for microdyalisis and behavioral test	25I-NBOMe induced wet dog shakes, and the effect was reduced by selective 5HT_2A_ and 5HT_2C_ antagonists, but not by selective 5HT_1A_ antagonist. 25I-NBOMe increased glutamate, DA and 5HT release and the effect was blocked by selective 5HT_2A_ and 5HT_2C_ and reduced by a selective 5HT_1A_ antagonist.	[52]
Wistar-Han rats	25I-NBOMe (0.3 mg/kg/day for 7 days; s.c.) for microdyalisis and behavioral tests	25I-NBOMe increased levels of DA, 5HT, GLU, and Ach in the frontal cortex weakly with respect to acute injection. It enhanced DA and 5HT levels in the striatum and nucleus accumbens more deeply than acute injection. It increased Ach levels in the frontal cortex, striatum and nucleus accumbens. 25I-NBOMe induced a reduction in motor activity, impaired memory capability, and induced anxiety.	[53]
Wistar-Han rats	25I-NBOMe (0.3 mg/kg/day for 7 days)	25I-NBOMe decreased the number of astrocytes in the frontal cortex and mPFC and the number of microglia cells in the frontal cortex.	[54]
ICR (CD-1) mice	25I-NBOMe (0.001, 0.01, 0.1, 1, 10 mg/kg; i.p.)	25I-NBOMe induced a decrease in visual and acoustic responses and increased the reaction time. A 10 mg/kg dose induced a transient decrease in the total distance travelled. It altered the startle response (1 and 10 mg/kg) and disrupted the PPI (0.01–10 mg/kg).	[55]

## Data Availability

The data presented in this study are available upon request for researchers of academic institutes who meet the criteria for access to confidential data.

## Abbreviations

The following abbreviations are used in this manuscript:

25I-NBOMe	4-Iodo-2,5-dimethoxy-N-(2-methoxybenzyl) phenethylamine
LTD	Long-term depression
LTP	Long-term potentiation
mPFC	Medial prefrontal cortex
NAc	Nucleus Accumbens
NPSs	Novel Psychoactive Substances
PPI	Prepulse inhibition
